# Buildings’ Biaxial Tilt Assessment Using Inertial Wireless Sensors and a Parallel Training Model

**DOI:** 10.3390/s23115352

**Published:** 2023-06-05

**Authors:** Luis Pastor Sánchez-Fernández, Luis Alejandro Sánchez-Pérez, José Juan Carbajal-Hernández, Mario Alberto Hernández-Guerrero, Lucrecia Pérez-Echazabal

**Affiliations:** 1 Instituto Politécnico Nacional, Centro de Investigación en Computación, Juan de Dios Bátiz Ave., México City 07738, Mexico; 2 Electrical and Computer Engineering Department, University of Michigan, 4901 Evergreen Rd, Dearborn, MI 48128, USA; 3 Escuela de Arquitectura Arte y Diseño, Instituto Tecnológico y de Estudios Superiores de Monterrey, Monterrey 64849, Mexico; lucrecia_perez@tec.mx

**Keywords:** real-time measurement, structural health monitoring, biaxial tilt angle, inclination severity, building applications, signal processing, time-series algorithms

## Abstract

Applications of MEMS-based sensing technology are beneficial and versatile. If these electronic sensors integrate efficient processing methods, and if supervisory control and data acquisition (SCADA) software is also required, then mass networked real-time monitoring will be limited by cost, revealing a research gap related to the specific processing of signals. Static and dynamic accelerations are very noisy, and small variations of correctly processed static accelerations can be used as measurements and patterns of the biaxial inclination of many structures. This paper presents a biaxial tilt assessment for buildings based on a parallel training model and real-time measurements using inertial sensors, Wi-Fi Xbee, and Internet connectivity. The specific structural inclinations of the four exterior walls and their severity of rectangular buildings in urban areas with differential soil settlements can be supervised simultaneously in a control center. Two algorithms, combined with a new procedure using successive numeric repetitions designed especially for this work, process the gravitational acceleration signals, improving the final result remarkably. Subsequently, the inclination patterns based on biaxial angles are generated computationally, considering differential settlements and seismic events. The two neural models recognize 18 inclination patterns and their severity using an approach in cascade with a parallel training model for the severity classification. Lastly, the algorithms are integrated into monitoring software with 0.1° resolution, and their performance is verified on a small-scale physical model for laboratory tests. The classifiers had a precision, recall, F1-score, and accuracy greater than 95%.

## 1. Introduction

Cities in lacustrine and seismic zones face a significant challenge; their buildings are subject to predisposing agents and triggers of various damages. Underground water extraction changes ground subsidence and its dynamic properties; these effects are relevant during an earthquake, such as those found in the Mexico City Valley [1]. Ground subsidence can cause building biaxial tilts, which can become critical with serious safety risks. Permanent monitoring and structural health monitoring (SHM) [2,3,4,5,6] allow the acquisition of time series with historical data, including their behavior before and after seismic events. Differential settlements, together with the structural characteristics of the building, can cause uneven slopes in its walls [7,8,9]. These tilt patterns can be diverse, and the value of their angles allows for establishing their severity levels. Measurements in real time show the importance of damage detection and building assessment by placing sensors and assuming weak areas. This framework is considered in evaluation and self-monitoring [10,11], including structural health monitoring for restoring heritage buildings [12,13].

Multidisciplinary efforts and new technologies have allowed the development of high-performance monitoring systems. Structural health monitoring implements a damage identification strategy for infrastructure [14,15,16], including tilts and high-rise building safety detection methods [17]. The approach consists of integrating technologies that allow obtaining information for the prevention of injuries in structures, such as vibrations detected in towers [18,19] and bridges [20]. Some studies used the dynamic acceleration produced by inertial sensors according to the application requirements [21]. However, they did not include algorithms for time-series processing, frequent inclination pattern recognition in areas with differential settlements, or a distributed architecture based on Wi-Fi technologies and client/server systems.

This work uses noisy static acceleration, unlike others where dynamic acceleration is essential, such as vibration analysis [22] and biomechanical tremor measurement [23]. In [24,25], a case study based on a wireless sensor network was analyzed; however, algorithms for the time-series treatment of static acceleration, inclination patterns, and pattern recognition were not presented. The authors of [24] presented only a short review of structural health monitoring of historic buildings. Other papers addressed the monitoring of electrical towers [25,26] with wireless systems or vibration inspection systems for offshore platform structures [27]. Some projects analyzed injuries due to telluric movements, evaluating the performance of prototypes during and after earthquakes [28,29]. Others used modeling methods and frameworks for early warning systems [30]. 

Other structural health monitoring strategies [31,32,33] have been presented; nevertheless, they are not based on permanent online monitoring systems for multiple buildings. Likewise, biaxial tilt patterns and their evolution due to differential soil settlement and random events, such as earthquakes, are not considered.

The number of sensors may vary depending on the work focus and type of measured variables, from monitoring a tower with more than 100 sensors to robust systems with more than five [34]. Microelectromechanical systems (MEMS) technology represents a significant advantage due to cost reduction, as presented in [5,6,17,29,35]; likewise, their reduced sizes are more widely accepted for optimizing nonintrusive monitoring in buildings. Many works have been developed on wireless sensor networks that help create embedded systems for monitoring civil infrastructures [27,34,35,36].

Several methods can be implemented to establish which machine learning algorithms (MLA) are the most efficient for the application under development. For example, in [37], Bayesian optimization was conducted to tune the hyperparameters of each MLA. The results indicated that support vector machines are the most accurate compared to the decision tree, naïve Bayes, discriminant analysis, and K-nearest neighbor for local damage detection in reinforced concrete bridges. In other cases, structural damage detection based on data-driven techniques using a deep neural network was proposed to study a large in-orbit flexible system. The model was trained using the sensor-measured time-series responses [38] and a distributed network of accelerometers [39]. 

Computational systems with distributed architecture in real time allow the simultaneous monitoring of several buildings in a control center. This paper presents a computational approach based on inertial sensors, as well as a parallel neural model for biaxial tilt pattern recognition and severity determination for rectangular buildings. Section 2 provides a general description of the distributed system, methods to improve the inertial measurement unit (IMU) signals, the proposed tilt patterns and parallel neural models for pattern recognition and tilt severity analysis, and their metrics. Section 3 presents a small-scale physical model for laboratory tests and a comparison with more closely related work. Lastly, the conclusions are given in Section 4.

## 2. Materials and Methods

### 2.1. Real-Time Measurements

**Figure 1:**
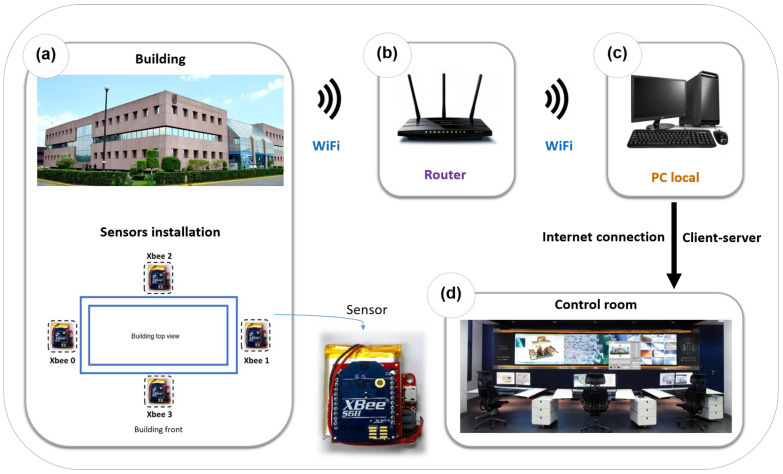
System overview. Sensor including IMU + the wireless communication module with the IEEE 802.15.4 protocol and a rechargeable battery. (**a**) Schematic of the four sensors installed on each of the walls of the rectangular building. (**b**) Local computer acquiring the sensors’ signals through a router. (**c**) Client–server architecture (TCP/IP protocol) of the system. (**d**) Monitoring of one or several buildings in a control room from any geographical location.

Figure 1 shows a general diagram of the system consisting of four inertial measurement units (IMUs) with MEMS technology. Each IMU incorporates an accelerometer, gyroscope, and magnetometer. The digital-output triple-axis accelerometer has a full-scale programmable range of ±2 g, ±4 g, ±8 g, and ±16 g with integrated 16 bit ADC. 

The digital-output triple-axis gyroscope has a full-scale range until ±2000°/s and integrated 16 bit ADC. The triple-axis silicon monolithic Hall-effect magnetic sensor has an output data resolution of 14 bit (0.6 μT/LSB) or 16 bit (15 μT/LSB) and a full-scale measurement range of ±4800 μT. Figure 1a shows a schematic of the four sensors installed on each of the walls of the rectangular building. Each sensor includes an IMU, a wireless communication module with the IEEE 802.15.4 protocol (WIFI-Xbee) to create a fast point-to-multipoint network and a rechargeable battery. 

A local computer acquires the sensor signals through a router, as shown in Figure 1b,c. The system uses a client–server architecture (TCP/IP protocol) to monitor one or several buildings in a control room from any geographical location (Figure 1d).

The sensors’ location reference considers the front of the building. This work calculates the inclination angle, assuming no vertical and horizontal wall deformations exist. According to the expert knowledge of structure and architecture specialists, the recommendation for this project stage was to place the sensors as high as possible on each wall. Likewise, the suggestion was applied to the small-scale physical model for laboratory tests.

Although outside the scope of this work, for measurements related to vertical and horizontal wall deformations, more than one sensor should be used per wall. The sensor network and software were designed considering possible project growth.

Cities in lacustrine and seismic zones can have many buildings with diverse damage, including biaxial inclinations. They are still habitable but require frequent inspections, which must be carried out in medium periods. The situation is even more critical after a seismic event. Therefore, each vulnerable building must have a diagnosis and inventory of its main damages, which still do not put its habitability at risk but require periodic supervision. The biaxial inclination is typical in buildings on soil with differential settlements. For this case, the experts evaluate them periodically using manual high-precision inclinometers. 

On the basis of IMU measurements, the calculated inclination for this work ranged from 0° to 1.5°, with a resolution of 0.1°. The proposed method allows a resolution of <0.1° depending on the used sensors. 

The model uses angles based on relative variables (deviation variables) concerning an initial inclination that could exist when the measurements and monitoring begin; there may be zero or nonzero initial slope in each of the four walls. This initial tilt information must be obtained by expert inspection before the real-time monitoring system begins to operate.

Additionally, accelerometer signals contain electrical noise, and the system can detect temporary vibrations. For such reasons, the selected sampling frequency is 50 samples per second (S/s) to apply digital filters with a cutoff frequency of up to 25 Hz. Furthermore, a quaternion-based orientation filter [40] for inertial sensors effectively separates static and dynamic accelerations. The time series of the measured inclinations is treated with a proposed algorithm of successive repetitions, which eliminates other disturbances still present. Section 2.2 describes the combination of these methods, which significantly improves the final results. Building inclinations evolve over long periods (months or years). This work verified different patterns and their evolution with computationally generated values and a small-scale physical model for laboratory tests, as described in Section 3.

### 2.2. Methods Combination for the Preliminary Treatment of Measurements

#### 2.2.1. Orientation Filter for Inertial Sensors

A useful accelerometer feature is the static acceleration measurement due to the Earth’s gravity, which is used to obtain the biaxial tilt of buildings. However, extensive tests showed that the dynamic acceleration signals existing in the sensors (caused by transient vibrations, fundamentally) are among the causes of interference with the calculated lean angles. It is necessary to obtain only the static acceleration. For this purpose, the orientation filter utilizes a quaternion representation. Accelerometer and magnetometer data are employed in an optimized gradient-descent algorithm to calculate the direction of the gyroscope measurement error as a quaternion derivative [40,41].

The quaternions are an extension of the complex numbers of four dimensions and can represent space rotations. They characterize the rigid body orientations or reference frames in three-dimensional space. For example, an orientation of the reference frame *B* relative to the reference frame *A* can be realized through a rotation of the angle *θ* around an axis $A_{\hat{r}}$ defined in frame *A* [41]. 

**Figure 2:**
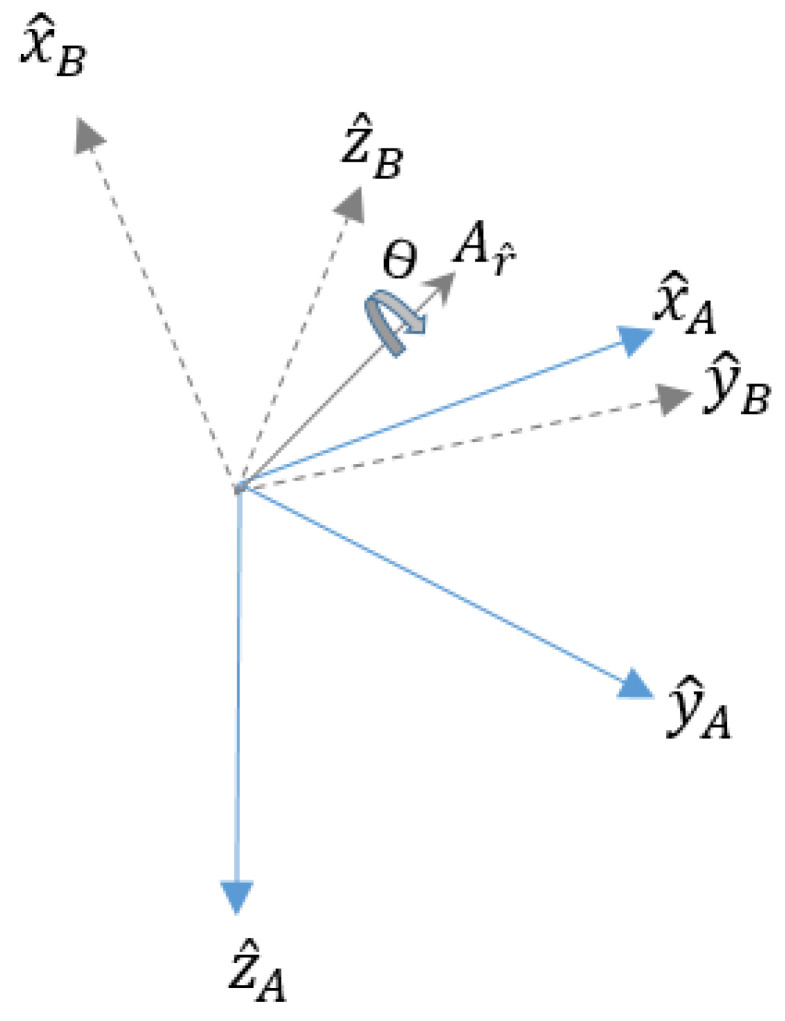
An orientation of frame *B* relative to frame *A* can be attained through a rotation of the angle *θ* around an axis $A_{\hat{r}}$ defined in frame *A*.

This is presented graphically in Figure 2, where the mutually orthogonal unit vectors ${\hat{X}}_{A}$, ${\hat{Y}}_{A}$, ${\hat{Z}}_{A}$ and ${\hat{X}}_{B}$, ${\hat{Y}}_{B}$, ${\hat{Z}}_{B}$ define the main reference frame axes *A* and *B*, respectively [41].

The quaternions that describe an orientation must be normalized and of unit length for an orientation q^BA defined in Equation (1).

(1) q^BA=q1 q2 q3 q4=(cosθ2) (−rxsinθ2) (−rysinθ2) (−rzsinθ2).

In this sense, the orientation filter is applied to the inertial sensor measurements. The sensor orientation is determined using Madwick’s algorithm in [41], and a representation is obtained using quaternion calculations, as shown in Equation (1).

Subsequently, the unitary vector of the Earth’s acceleration $\left[x,\;y,\;z\right]=\left[0,\;0,\;1\right]$ is rotated to the sensor position or IMU, obtaining its static acceleration at each sampling instant. 

**Figure 3:**
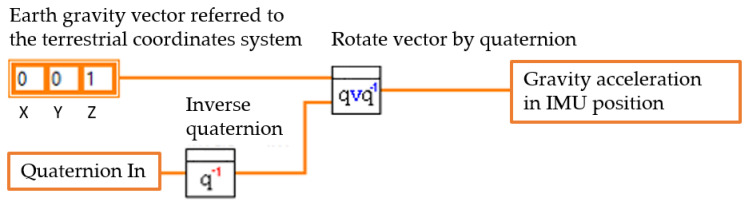
Quaternion rotation method to obtain gravity acceleration.

**Figure 4:**
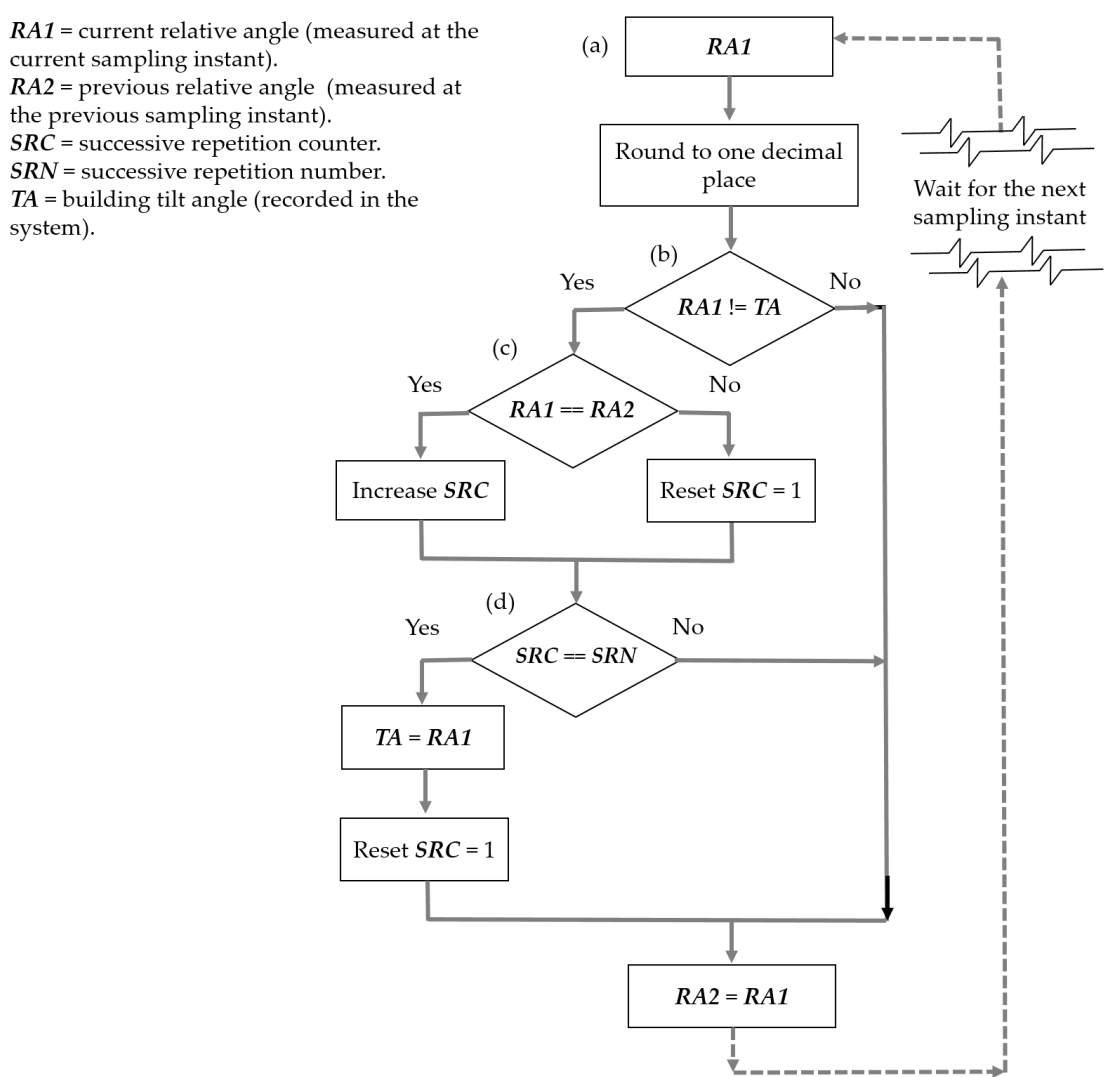
Algorithm block diagram of successive repetitions for a sampling instant and each of the eight biaxial tilt angles. The explanation of (**a**–**d**) may be read in the paragraph before Figure 4.

Figure 3 illustrates the quaternion rotation method to get gravity acceleration to calculate biaxial tilt angles. This calculation using the orientation filter eliminates any component of dynamic acceleration resulting from temporary vibrations in the building, less than 25 Hz. Any disturbance still existing in the signal will be treated in the time series using the successive repetitions algorithm proposed in Section 2.2.3, as illustrated by the block diagram in Figure 4.

#### 2.2.2. Average Filter in Real Time

This filter averages many input signal points to produce each output signal point. Equation (2) presents this definition.

(2) $$Y\left(k\right)=\frac{1}{n}\;\overset{k}{\underset{i=k-n+1}{\sum }}X\left(i\right),$$

where *n* is the number of samples to average, $X\left(i\right)$ is the filter input at sampling time i, and *k* is the current sampling time. Equation (3) shows the programmed recursive expression.

(3) $$Y\left(k\right)=Y\left(k-1\right)+\frac{1}{n}\left[X\left(k\right)-X\left(k-n\right)\right],$$

where $Y\left(k\right)$ represents filter output at sampling time k, $Y\left(k-1\right)$ is the filter output at sampling time k−1, $X\left(k\right)$ is the input to the filter at sampling time k, $X\left(k-n\right)$ represents the average filter input at sampling time k−n, and *n* is the number of samples to average.

The filter only requires input information at the current sampling time $X\left(k\right)$ and at the last *n* previous sampling times. The filter must be initialized, saving $Y\left(k\right)$, $Y\left(k-1\right),$ and the previous n inputs. The filter is efficiently programmed because the last *n* inputs are shifted, eliminating the oldest.

#### 2.2.3. Successive Repetitions Algorithm

Wind or heavy traffic can produce temporary building vibrations, affecting the real-time inclination angle measurements. Figure 4 presents an algorithm of successive repetitions based on a numerical value array, which processes the time series of biaxial tilt angles. This algorithm establishes that the inclination angle must maintain its value for several successive repetitions to accept that there has been a change in it; each repetition is considered at the corresponding sampling instant. 

This algorithm of successive repetitions is useful for diverse applications, and the successive repetition number can be adjusted to ensure stability and convergence. In this work, on the basis of numerous experimental tests, the successive repetition number is set to 200, and the results are reliable in their accuracy. The convergence time is always 4 s since the sampling rate is 50 S/s.

***RA1*** indicated in (a) is calculated after the IMU inertial signal processing by the filters of Section 2.2.1 (orientation filter) and Section 2.2.2 (average filter for real time). In (b), the system checks if ***RA1*** differs from the building tilt angle ***TA*** recorded in the system. If there are no differences, the system considers the building tilt angle (***TA***) unchanged, and only the current relative angle ***RA1*** is assigned to the previous relative angle ***RA2*** for processing in the next sampling instant. If in (b) the answer is yes, the processing is indicated in the block diagram. (c) If ***RA1*** is equal to ***RA2***, then ***SRC*** increases by one; otherwise, ***SRC*** = 1. (d) If ***SRC*** is equal to ***SRN***, the number of successive repetitions of the ***RA1*** angle value has occurred, and the system considers that the calculation of the current relative angle measured at the present sampling instant is stable, making ***TA*** *= **RA1***.

This processing is applied for each of the eight biaxial tilt angles in each sampling instant.

### 2.3. Tilt Patterns

**Figure A1:**
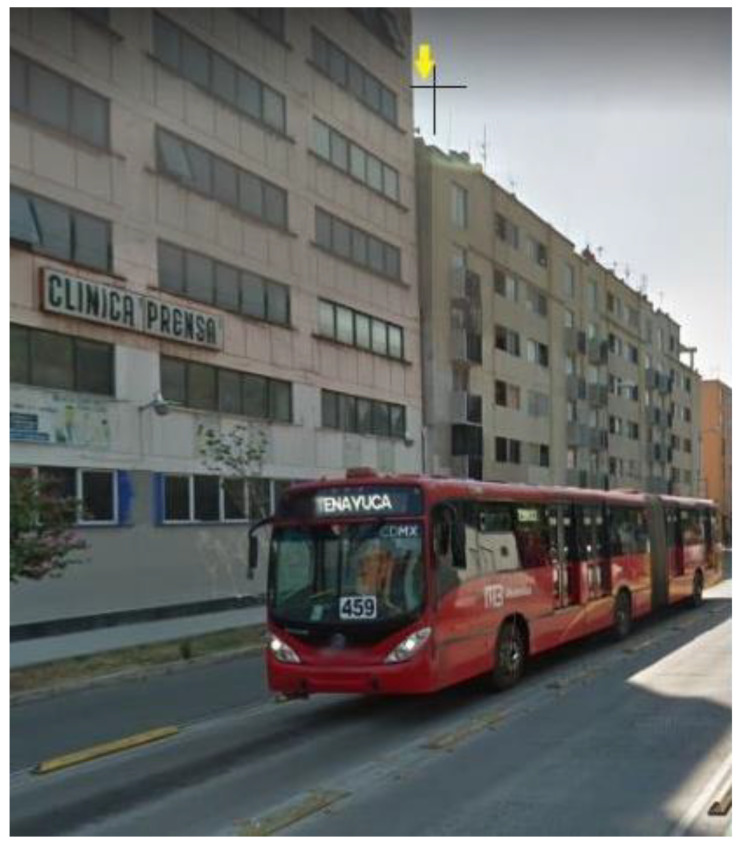
Clinic Prensa, which is a leaning building and is under supervision.

**Figure A2:**
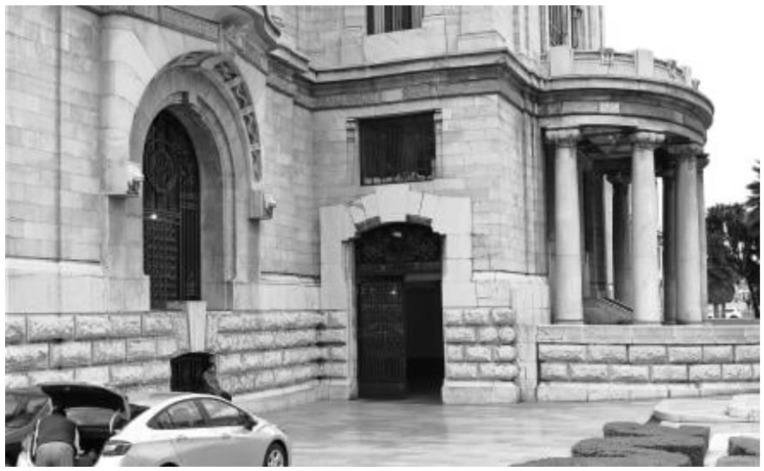
The Palace of Fine Arts in Mexico City.

Eighteen basic patterns were proposed, representing the shapes of frequent inclinations in buildings, mainly considering the differential settlements [7,8,9]. Several buildings are leaning (off-kilter), from Big Ben at just 0.26° to the Sturluson church tower in Germany at 5.19° [42]. In general, construction regulations establish the maximum limits of the average inclination of the building through formulas based on its height, specified in percentages [43]. For example, the maximum slope allowed for a building 18 m high is 0.65% or 0.37°; for 36 m height, it is 0.48% or 0.28°. However, quite a few buildings have slopes greater than 1° and are still inhabited, although they are under supervision, as shown in Figure A1 (Appendix A) for the Mexico City Press Clinic and Figure A2 for the Palace of Fine Arts.

This work uses sexagesimal degrees; the tilt resolution is 0.1°, and the severity or range is from −1.5° to 1.5°. The tilt patterns and neural models developed can be applied to smaller resolutions, i.e., hundredths of a degree. Likewise, a conversion can be made to a percentage concerning the height of the building. The base patterns can be illustrated graphically by considering the inclinations’ general shapes. The tilt resolution of this project was 0.1°.

**Figure 5:**
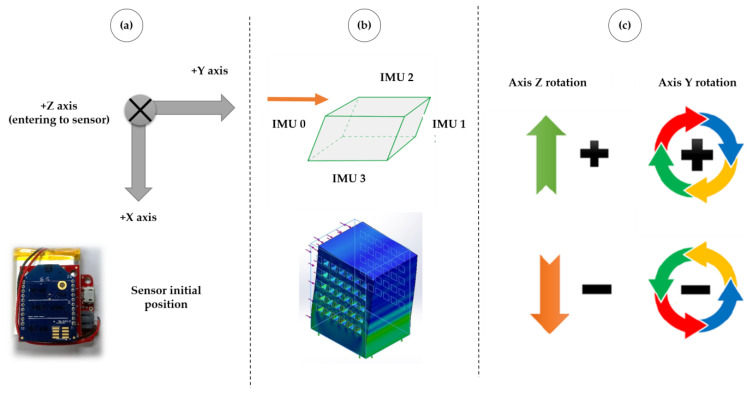
(**a**) Reference system for the sensors. (**b**) Example of pattern 0. (**c**) Angle rotation.

Figure 5a presents the sensors’ initial reference system (when installed on the walls). Two axes are used to measure the building’s tilt. The Y–Z plane represents the biaxial tilt, allowing the IMUs to be easily powered regardless of their location on the building walls. A generic building illustration is presented regarding the walls’ inclination. Figure 5b illustrates pattern 0, where all four walls tilt to the right. Figure 5c shows the rotation of the axes Y and Z, regardless of their location on the building walls.

**Figure 6:**
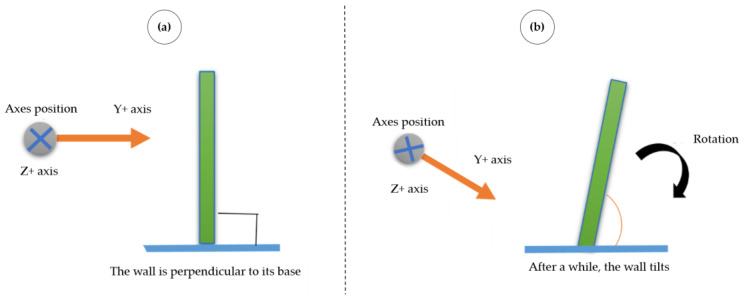
The possible evolution of the tilt angles. (**a**) The wall is perpendicular to its base, and there are no tilts. (**b**) The wall has tilted over time; the Y-axis is tilted clockwise and is represented by a positive sign.

Figure 6 illustrates an example of the wall’s possible tilts from its front view. The positive Y-axis is to the right, and the positive Z-axis goes into the page (to the sensor). In Figure 6a, the wall is perpendicular to its base, and there are no tilts. In Figure 6b, the wall has tilted over time; the Y-axis is tilted clockwise and is represented by a positive sign. 

**Figure 7:**
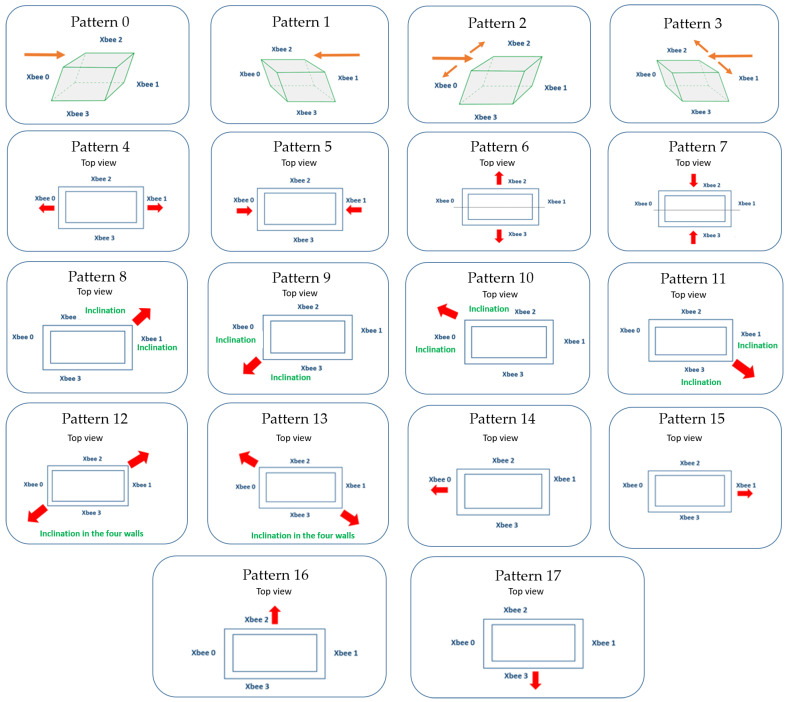
A simplified scheme of the 18 base patterns proposed in this work.

Figure 7 shows a simplified scheme of the 18 base patterns proposed in this work. Considering the expert knowledge of structure and architecture specialists, the 18 base patterns can cover the building tilt forms in practice and even more. These patterns contemplate each wall as a single entity; however, each wall tilt has a close relationship throughout the structure. Furthermore, if the classifier does not recognize a predominant pattern, the biaxial inclination of each wall is recorded independently by the real-time monitoring system. Likewise, the possible future redesign of the system may include new tilt types with classifier retraining and some changes to the model or includes more sensors per wall.

The biaxial tilt measured by the sensors is determined as shown in Equation (4), e.g., when the Z-axis has not changed.

(4) $$i_{w}=\left(g_{Y}^{w},\;g_{Z}^{w}\right)\;|\;g_{Z}^{w}=0,$$

where $i_{w}$ represents the biaxial inclination of the wall *w* of the building.

The ordered pair ($g_{Y}^{w},\;g_{Z}^{w}$) represents the relative angles in the Y- and *Z*-axes, respectively, for the wall *w | w* = 0 … 3. For example, for pattern 0, considering the resolution of 0.1°, Equation (5) is obtained for a wall 0.

(5) $$i_{0}=\left(g_{Y}^{0},\;g_{Z}^{0}\right)∴\;i_{0}=\left(0.1,\;0\right).$$

Equation (6) represents the building’s biaxial tilt patterns mathematically for its four walls.

(6) $$p_{x}=\left[i_{0},\;i_{1},\;i_{2},\;i_{3}\right]=\left[g_{Y}^{0},\;g_{Z}^{0},g_{Y}^{1},\;g_{Z}^{1},g_{Y}^{2},\;g_{Z}^{2},g_{Y}^{3},\;g_{Z}^{3}\right]|\;x=0\ldots 17,$$

where $p_{x}$ is the set of base tilt patterns, x is the base pattern,$\;i_{0},\;i_{1},\;i_{2},\;\mathrm{and}\;i_{3}$ are the tilt angles of walls 0, 1, 2, and 3, respectively, and $g_{Y}^{w},\;g_{Z}^{w}$ represent the relative angles in the Y- and Z-axes, respectively, for the wall *w | w* = 0 … 3.

The 18 base patterns were created using Equations (4) and (5). Equations (7)–(10) show examples for patterns 6 and 14 from Figure 7. Pattern 6 has inclinations in walls 2 and 3, as shown in Equation (7), with the rotation directions of Figure 5c.

(7) $$p_{6}=\left[g_{Y}^{0},\;g_{Z}^{0},g_{Y}^{1},\;g_{Z}^{1},g_{Y}^{2},\;g_{Z}^{2},g_{Y}^{3},-g_{Z}^{3}\right].$$

The values are substituted into Equation (7) considering the system resolution of 0.1°, resulting in Equation (8).

(8) $$p_{6}=\left[0,\;0,\;0,\;0,\;0,\;0.1,\;0,-0.1\right].$$

Pattern 14 presents tilt in wall 0, as shown in Equation (9). The values are substituted in Equation (10).

(9) $$p_{14}=\left[-g_{Y}^{0},\;g_{Z}^{0},g_{Y}^{1},\;g_{Z}^{1},g_{Y}^{2},\;g_{Z}^{2},g_{Y}^{3},\;g_{Z}^{3}\right].$$

(10) $$p_{14}=\left[-0.1,0,\;0,\;0,\;0,\;0,\;0,\;0\right].$$

Table 1 shows examples of patterns 0–3 of the four walls based on Equations (4)–(6).

So far, the example patterns have been for the minimum inclination of 0.1°. For the computer model’s training, variations of the patterns were made. For example, the pattern 0 for wall 0 illustrated with Equation (5), based on increments of 0.1°, can go up to $i_{0}=\left(1.5,\;0\right)$; the same applies for walls 1, 2, and 3. However, there may be differences in the inclination between the walls, which would result in a distorted pattern 0, but the computer system indicates the angles in each wall so that an expert has additional information on the base pattern recognized. Similar behavior can be presented for other patterns.

### 2.4. General Diagram of the Classification Model

**Figure 8:**
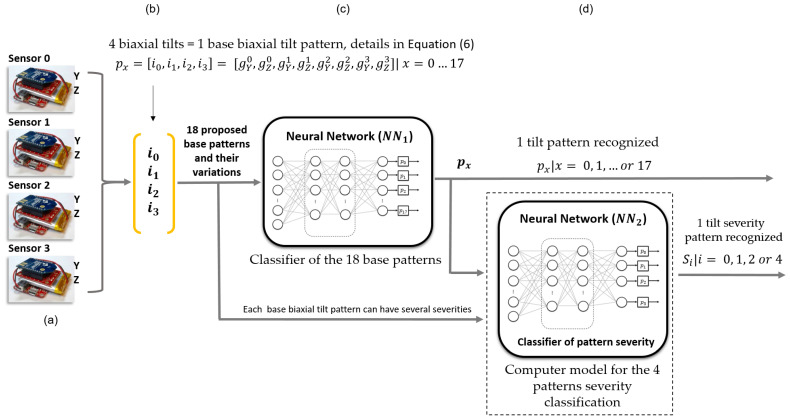
General diagram of the classification model. (**a**,**b**) Show the four sensors and the four biaxial tilts, respectively. (**c**) The classifier of base patterns. (**d**) The computer model for the pattern severity classification.

The model uses artificial neural networks for base tilt pattern recognition and its severity classification. Figure 8 illustrates the general diagram of the classification model. Figure 8a,b shows the four sensors and the four biaxial tilts, respectively. Figure 8c is the classifier of base patterns. Figure 8d is the computer model for the pattern severity classification, which includes the neural network $NN_{2}$ and an algorithm, which is detailed in Section 2.6.1.

$NN_{1}$ of Figure 8c is a feedforward neural network that uses the vector of biaxial tilts to classify the corresponding base pattern. The output is the classified base pattern $p_{x}$ (among the 18 proposed). The subsequent sections evaluate the performance of the classification models in Figure 8.

### 2.5. Classifier of Base Biaxial Tilt Patterns ($NN_{1}$) of Figure 8c

A multilayer perceptron neural network was used to classify the base tilt patterns. Parameters that are not directly learned within the neural network training were adjusted on the basis of exhaustive search-generated candidates from a grid of parameters (*grid-search*) [44,45]. This grid consisted of different values for each parameter, such as epochs, learning rate, and loss function. For fitting the model to the data, the possible combinations of the grid were evaluated, and the best combination was chosen according to a specific metric (in this case, accuracy). 

After applying the *grid-search* method, the chosen hyperparameters during the training phase were the mean squared error (*MSE*) as the loss function, learning rate α=0.0001, and training for 2000 epochs. The hidden layer and the output layer used a hyperbolic tangent activation function. 

A stratified cross-validation of the 18 features (base patterns) was implemented to obtain the training and a test subset (with a ratio of 70:27, respectively). The validation uses 3% of the dataset. Afterward, the neural network training was conducted using only the training subset, leaving the test subset out of the training phase. 

**Figure 9:**
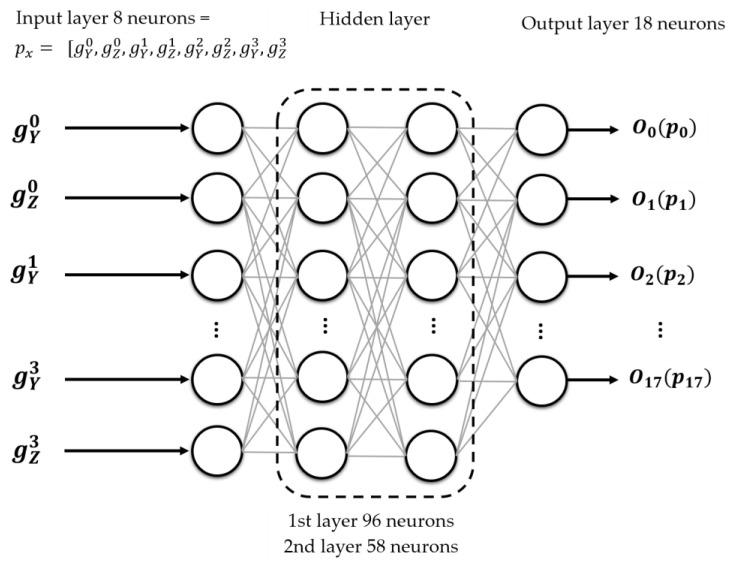
General schema of the classifier of base biaxial tilt patterns ($NN_{1}$).

To have more certainty that the neural network $NN_{1}$ architecture had learned adequately, the test subset was used as input for the neural network $NN_{1}$ in the test or evaluation phase of the classifier. The metric *MSE* was used to compare the trained neural network $NN_{1}$ performance. The coefficient of determination $R^{2}$ did not add different information. Through many tests, the topology shown in Figure 9 was selected.

$NN_{1}$ had eight input neurons, where ($g_{Y}^{0}\;,g_{z}^{0},g_{Y}^{1}\;,g_{z}^{1},g_{Y}^{2}\;,g_{z}^{2},g_{Y}^{3}\;,g_{z}^{3})$ represent the four ordered pairs of inclinations in the Y- and Z-axes for the four building walls. It had two hidden layers (96 and 58 neurons, respectively) and a layer of 18 output neurons to classify the 18 base patterns. According to a set of tests and the range of inclinations to be monitored for this work (−1.5° to 1.5°), the hyperbolic tangent activation function ($\gamma_{m}$) was used to process each of the outputs ($O_{m}$) with *m* = 0, …, 17.

For the neural network supervised training, a target output vector was related to each input vector, as shown in Equation (11).

(11) $$v_{x}=\left[p_{x},s_{x}\right],$$

where $v_{x}$ is the training dataset consisting of the base tilt pattern $p_{x}$ and their respective target vectors $s_{x}$. For the simulation, a computational algorithm generated $v_{x}$, varying the inclinations ±0.1° up to ±1.5°, according to the corresponding base pattern (0…17). A total of 12,900 patterns were used (approximately 715 patterns for each base pattern): 70% for training the network, 3% for validation, and 27% for testing, with which the best results were obtained using the library of Python neural networks.

Only one of the 18 output neurons is triggered when pattern recognition is excellent. In other cases, more than one is activated, but a predominant value indicates the pattern with the most remarkable similarity. This behavior, regarding the most notable similarity, is essential for future work improvements. In that sense, if a building presents a different tilt pattern than the 18 proposed in this work or one that is the combination of two or more base patterns or their distortions, then specialists can analyze the inclination angles returned by the measurement system for each wall. We propose a buildings’ biaxial tilt classification assessment with a computer expert system that can be progressively enriched with new scientific and technological contributions and the experience derived from its application in real buildings in each geographical area. For example, Mexico City has land with very particular characteristics.

**Figure 10:**
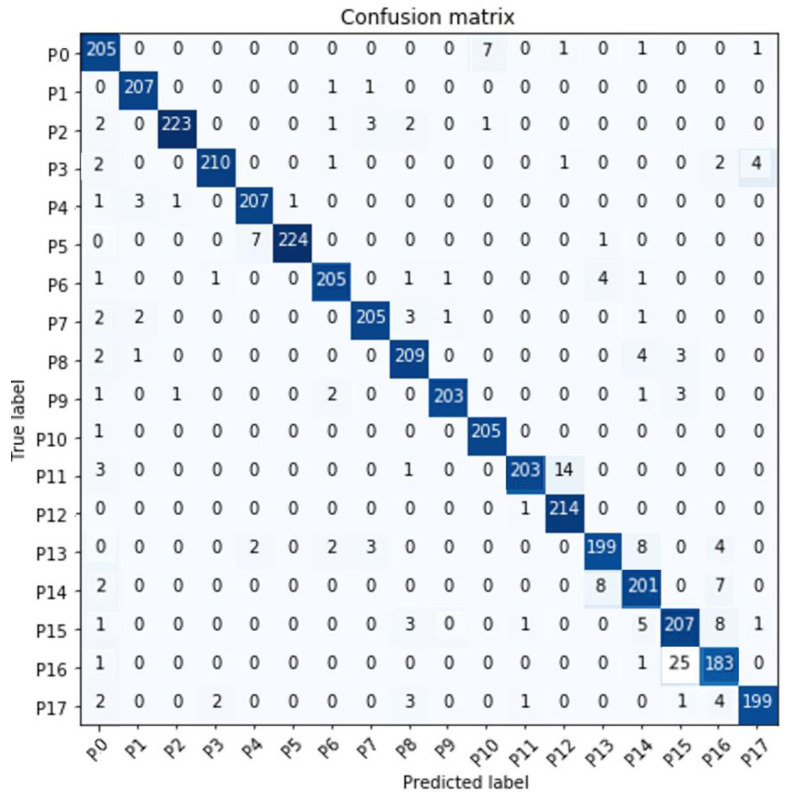
Confusion matrix to evaluate ***NN*_1._** The Y-axis represents the accurate classification assigned to the patterns (true label), while the X-axis indicates the classification given by the network (predicted label). The diagonal values show the correctly labeled patterns; the neural network classified them as the same pattern to which they belong. Table 2 shows the performance metrics of the multiclass classifier using macro-average, with values truncated to two decimal places.

Figure 10 presents the confusion matrix generated by the base pattern classification of $NN_{1}$.

**Table 2:** Performance scores of $NN_{1}$.

Metrics	Evaluation
Precision	0.95
Recall	0.94
F1	0.95
Accuracy	0.95

The precision [46] is the ratio shown in Equation (12).

(12) $$Precision=\frac{tp}{\left(tp+fp\right)},$$

where tp is the number of true positives, and fp is the number of false positives. The precision is intuitively the ability of the classifier not to label as positive a sample that is negative. The worst value is zero, and the best is one. *The recall* [46] is defined in Equation (13).

(13) $$Recall=\frac{tp}{\left(tp+fn\right)},$$

where tp is the number of true positives, and fn is the number of false negatives. Recall is intuitively the ability of the classifier to find all the positive patterns.

The $F_{1}$ score is interpreted as a weighted average of precision and recall, where an F1 score reaches its best value at one and worst score at zero. The relative contribution of precision and recall to the $F_{1}$ score is equal. The formula for the $F_{1}$ score is shown in Equation (14).

(14) $$F_{1}=2\times \frac{precision\times recall}{precision+recall}.$$

In the multiclass and multilabel case, this was the average of the $F_{1}$ score of each class with weighting depending on the average parameter.

Considering that some of the 18 base biaxial tilt patterns proposed can be infrequent in buildings, the classifier of base biaxial tilt patterns ($NN_{1}$) is very recommendable since the performance scores were higher than 94%. Section 3 presents examples of tilt pattern classification on a small-scale physical model for laboratory tests.

### 2.6. Tilt Severity Classification 

Each pattern (vector of eight angles) was labeled with an algorithm assigning the tilt severity according to Equation (15).

(15) $$S=\left\{\begin{array}{c} Reduced\;risk\;0<g\leq 0.4\\ High\;risk\;0.4<g\leq 0.8\\ \begin{array}{c} \;Serious\;risk\;0.8<g\leq 1.2\\ Critical\;risk\;1.2<g\leq 1.5 \end{array} \end{array},\right.$$

$$g=g_{Y}^{w}\;or\;g_{Z}^{w}|w=0\;\ldots \;3\;\exists \;p_{x},$$

where *S* is the tilt severity, g represents a relative angle in any Y- or Z-axis for a wall (see Equation (6)), and $p_{x}$ is the set of base biaxial tilt patterns.

The eight tilt angles can frequently have similar magnitudes because each wall has a close relationship throughout the structure; this is considered in the training patterns. In this way, when the severity labeling algorithm detects an angle within $p_{x}|\;x=0\ldots 17$ exceeding some limit of the four intervals presented in Equation (15), the severity pattern is labeled with the upper level. 

**Figure 11:**
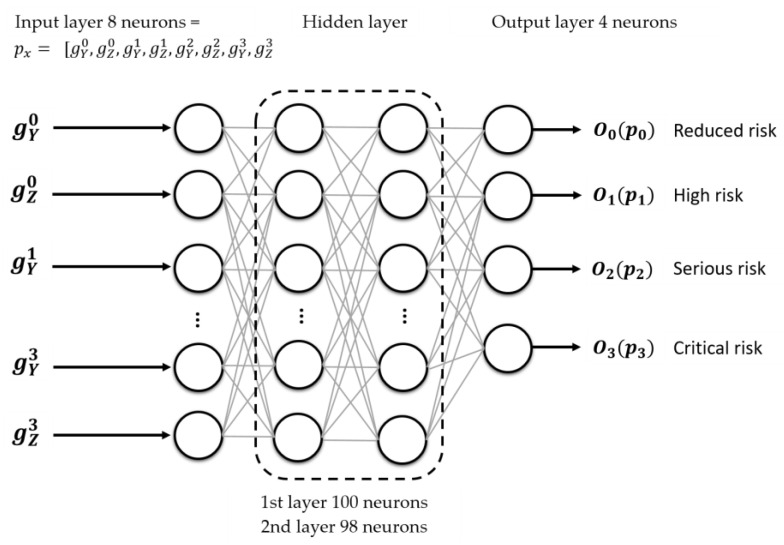
Multilayer perceptron neural network (*NN*_2_) topology to classify biaxial tilt severity.

Experimental work began with various topologies of a multilayer perceptron neural network to evaluate its performance as a classifier of the tilt severity or to consider other options. Figure 11 shows the selected topology with eight inputs ($g_{Y}^{0}\;,g_{z}^{0},g_{Y}^{1}\;,g_{z}^{1},g_{Y}^{2}\;,g_{z}^{2},g_{Y}^{3}\;,g_{z}^{3}$); two hidden layers (100 and 98 neurons, respectively) and an output layer for the severity classification. A hyperbolic tangent function was used as the activation function ($\gamma_{m}$), to process the outputs ($O_{m}$). Table 3 shows how the neurons will be activated.

The first option for training the model to classify tilt severity used *a single dataset from the 18 base biaxial tilt patterns*; however, the differences in the complexities between the base patterns produced significant distortions. A model named parallel was implemented.

#### 2.6.1. Parallel Recognition Model to Classify the Tilt Severity

**Figure 12:**
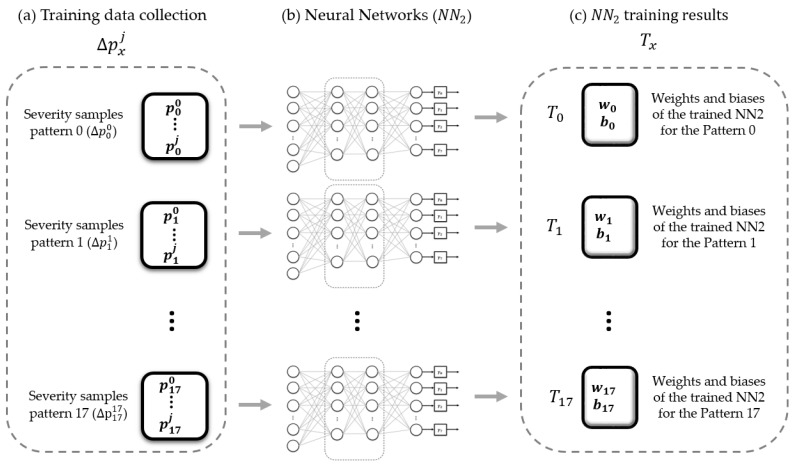
Parallel recognition model training.

This model was specialized for recognizing each base pattern severity. Figure 12 presents the schema of the parallel recognition model training. It was trained with 18 datasets, each one formed by the severity patterns of a single base pattern. 

The network was trained with 18 datasets, as shown in Equation (16).

(16) $$\Delta p_{x}=\Delta p_{x}^{j},|x=0\;\ldots \;17,$$

where $\Delta p_{x}$ is a set with the number *j* of severity variations based on the pattern *x*.

Depending on its complexity, the number *j* must be different for each base pattern; more complex base patterns (different tilt angles on each wall) will have greater variations. The parallel recognition model training of Figure 12 allows such differences in the number of variations in tilt severity between base patterns without implications on possible training data imbalances. Figure 12a illustrates that severity samples for patterns 0–17 can generally be different. Depending on the complexity of the base pattern, 569–3884 samples were used for each training set (18 sets): 70% for training, 3% for validation, and 27% for testing.

**Figure 13:**
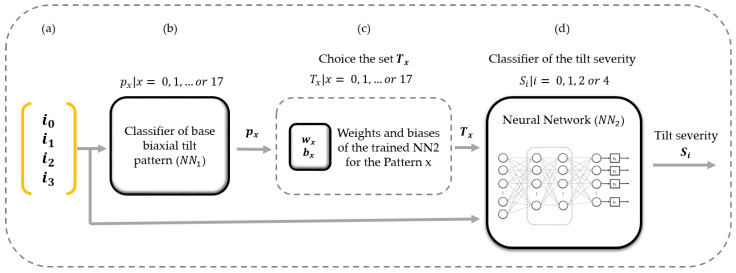
The classifier of the base biaxial tilt pattern and parallel recognition model implementation to classify the tilt severity. (**a**) The input of the four biaxial tilts, illustrated initially in Figure 8b. (**b**) The classifier of base biaxial tilt patterns of Figure 8c. (**c**) This output allows the choice of a set of weights and biases (see Figure 12c). (**d**) The classifier of the tilt severity (see Figure 11).

Figure 13 presents the base biaxial tilt pattern classifier and parallel recognition model implementation to classify the tilt severity. Figure 13a is the input of the four biaxial tilts, illustrated initially in Figure 8b. Figure 13b is the classifier of base biaxial tilt patterns of Figure 8c, which outputs a base pattern $p_{x}|x=0,\;1,\;\ldots \mathrm{or}\;17$. This output allows the selection (Figure 13c) of the set $T_{x}|x=0,\;1,\;\ldots \mathrm{or}\;17$ with the weights and biases of the trained ***NN*_2_** indicated in Figure 12c. Lastly, Figure 13d is the classifier of the tilt severity (see Figure 11), which has an output $S_{i}|i=0,\;1,\;2,\;\mathrm{or}\;4$, corresponding to reduced risk, high risk, serious risk, or critical risk, respectively. 

**Figure 14:**
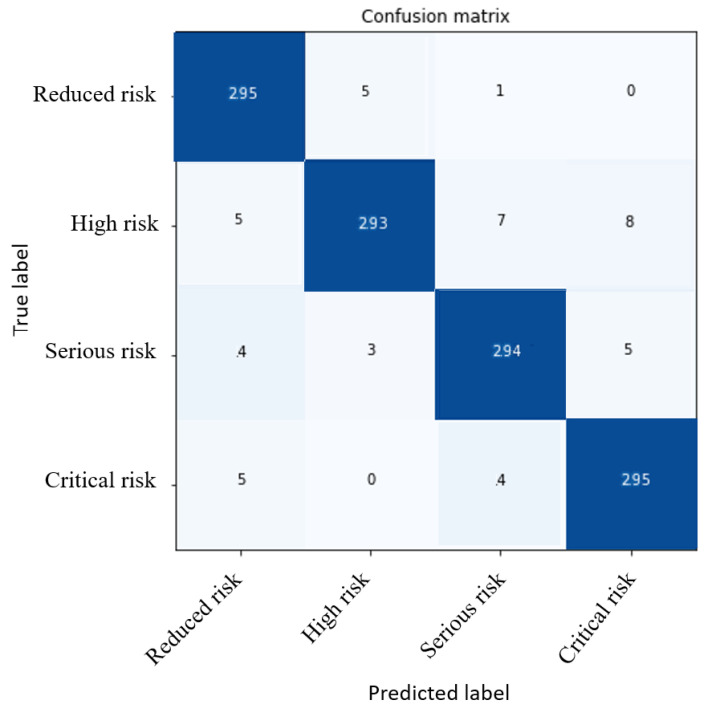
Most representative confusion matrix of ***NN*_2_**.

Figure 14 presents the most representative confusion matrix generated by the parallel recognition model to classify the tilt severity. Table 4 shows its metrics. The tests were performed with the severity samples of each of the 18 base tilt patterns. The classifier metrics were between 0.94 and 0.98, depending on the complexity of the base tilt pattern. The specialization based on the training of each neural network is illustrated in Figure 12, and the implementation is shown in Figure 13. The performance was high, eliminating possible ambiguities between different patterns. The neural network was specialized to recognize the specific severity of each base pattern. 

## 3. Results and Discussion

**Figure 15:**
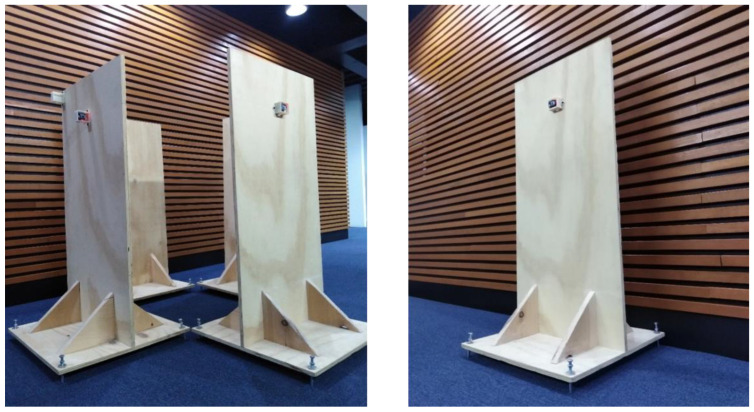
A small-scale physical model for laboratory tests in rectangular buildings.

Figure 15 presents a small-scale physical model for laboratory tests. It was built to verify the distributed measurement system and the tilt pattern recognition in rectangular buildings. It comprised four separate pieces that simulated each of the walls.

**Figure 16:**
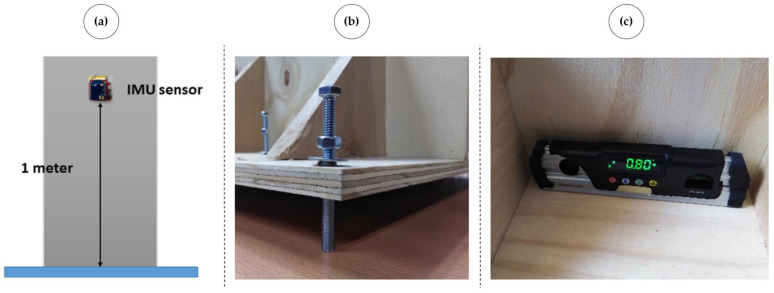
(**a**) IMU installation schematics. (**b**) Screws for tilt simulation. (**c**) Digital inclinometer.

Figure 16a shows the installation scheme of the IMUs. The sensors were located in the center, 1 m from the base, for the tests. Each wall had a mechanism based on screws at the corners of their respective bases, as shown in Figure 16b, to generate the base patterns and their corresponding severities. The digital inclinometer shown in Figure 16c permitted verifying that the distributed measurement system based on the IMUs correctly detected the angles generated in the prototype from an initial setting (initial reference). We emphasize that the model used angles based on relative variables (deviation variables) concerning an initial inclination that could exist when the measurements and monitoring began; there could be zero or nonzero initial tilt in each of the four walls. This initial tilt information can be obtained by expert inspection before the real-time monitoring system begins to operate.

Numerous laboratory tests in the small-scale physical model for rectangular buildings of Figure 15 were conducted to verify the base pattern classification and severity. Table 5 shows 96 examples of the base pattern classification and their variations. Inclination patterns in the prototype were calibrated using a digital inclinometer with two decimal places. The accuracy was 100% because the real patterns in the physical model had little distortions compared with the definitions of Figure 7. If there were failures to recognize some patterns highly distorted concerning the proposed base patterns, the measurement system returned the inclination angles in each of the eight axes. The patterns were produced in the physical model, considering the building as a single entity; the tilts in each wall had a close relationship throughout the structure. Distorted patterns are marked with asterisks, indicating the distortion for each axis.

Likewise, many tests were performed to classify the inclination severity level on the basis of the relationship presented in Equation (15) and Table 3. The ***NN*_2_** model activated a neuron with a higher numerical value. Table 6 shows 30 examples of the most relevant results. Table A1 of Appendix B presents additional examples. Severities were configured in the physical model (prototype) with calibrated angles using the digital inclinometer with two decimal places. Of the 30 tests performed, one was misclassified as reduced (number 12) since its severity was close to high according to Equation (15). The small-scale physical model in rectangular buildings allowed experimenting with very different tilts between the walls; that is, while one wall had a more significant inclination, another had an angle close to 0°, which can be infrequent in real buildings. In the future, this behavior could improve the definition of severities by evaluating the non-inclusion of some mathematically subtle differences.

There are more closely related studies for building monitoring. Acceleration response functions were presented in [6], which is one of the most popular options in the structural health monitoring field. However, tilt patterns and their severity were not recognized. A design and validation of a scalable and reconfigurable interesting structural health monitoring system [5] was based on triaxial accelerometers in microelectromechanical systems. It computes the frequency response functions for the subsequent modal analysis based on efficient hardware and graphical programming environment, albeit not inexpensively. Likewise, that work did not consider biaxial tilt patterns, their severity, and their evolution in real time.

Another extensive and detailed study introduced a convolutional neural network that exploits a form of measured compressed response data through transfer learning-based techniques [32]. Although damage patterns were presented, tilt patterns and their severity were not established, which is requested in urban areas with differential soil settlements. In [47,48], MEMS technology accelerometers were used to monitor inclination, the data were transmitted wirelessly, and the tilts of two axes were analyzed; however, in both works, patterns were not classified, and tilt severity was not determined.

In [25], tilts were monitored with MEMS accelerometers in real time, and a user interface was created; six sensors were installed on the structure body to monitor three axes, but tilt patterns were not recognized, and their severity was not calculated. In some projects, real-time monitoring was carried out [18,25]; however, alarms were only included in [3]. In [49], a system with accelerometers and inclinometers for structural health monitoring was presented but not based on permanent supervision.

**Figure A3:**
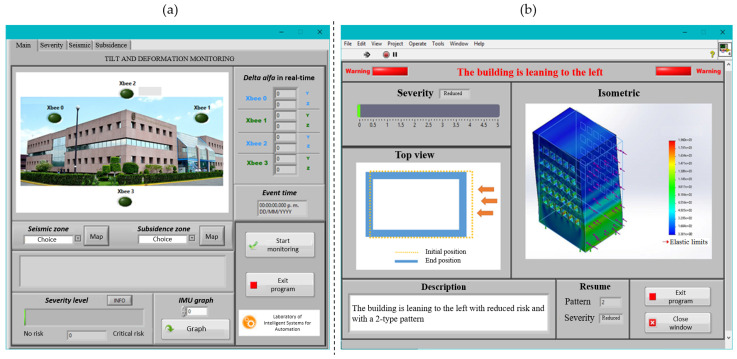
(**a**) The main screen of the user interface. (**b**) A pop-up alarm screen.

In this work, permanent monitoring allowed information processing for tilt angle visualization in real time. A user interface is presented in Figure A3a as an example. In addition, the software allows observing the sensor status, the timestamp of the recorded phenomena, and the tilt severity levels. When the system detects an inclination pattern, the machine learning models define the activation of emerging alarms; for example, Figure A3b provides the most relevant information on the detected pattern and its severity.

## 4. Conclusions

A wireless real-time computational system was developed using a distributed architecture and a TCP/IP server client, with inertial sensors to measure and analyze biaxial tilt signals in rectangular buildings. This work effectively combined a set of tools for treating inertial measurements, and a new algorithm of successive numeric repetitions was developed with successful results. It can be used in various applications to improve the quality of digital signals acquired and avoid false alarms due to the presence of unwanted oscillations.

The neural models allowed recognition of the biaxial tilt patterns and their severity in four walls. The classifiers were verified with a small-scale physical model for laboratory tests in rectangular buildings; their good performances were documented in the paper. Recognition metrics were above 95%. The system could be applied successfully since the recognition errors occurred for very infrequent patterns in real buildings. A parallel tilt severity training and recognition model was developed on the basis of specialization for each base pattern, depending on its complexity.

As the building tilt progress can occur over months or years or when an earthquake happens, the method’s feasibility must be verified first on a small-scale physical model for laboratory tests. An immediate goal for this work will be its application to real vulnerable buildings and an improvement the system based on fieldwork. Furthermore, using a more significant number of sensors for each wall, fully supported by the hardware and software, would allow for classifying other forms of inclination and deformation. This is an open research and development field where multidisciplinary researchers and engineers can contribute and enrich the scientific and technological state of the art. Machine learning tools can be integrated with low-cost wireless technology and frequently used software.

For the necessary continuous improvements of building biaxial tilt assessment models, multiclass receiver operating characteristic (ROC curves) and their respective area under the curve (AUC) may be presented over a range of thresholds, differentiating the performance from random guessing, i.e., one-vs.-rest multiclass ROC. This could help to optimize the detection algorithm to achieve the highest level of accuracy in detecting damage while minimizing false positives.

## Figures and Tables

**Table 1:** Examples of tilt angles of patterns 0–3.

	IMU 0 (Wall 0)	IMU 1 (Wall 1)	IMU 2 (Wall 1)	IMU 3 (Wall 3)
Pattern 0				
$\mathrm{Delta}\;Y\;(g_{Y})$	0.1	0.1	0.1	0.1
$\mathrm{Delta}\;Z\;(g_{Z})$	0	0	0	0
Pattern 1				
$\mathrm{Delta}\;Y\;(g_{Y})$	−0.1	−0.1	−0.1	−0.1
$\mathrm{Delta}\;Z\;(g_{Z})$	0	0	0	0
Pattern 2				
$\mathrm{Delta}\;Y\;(g_{Y})$	0.1	0.1	0.1	0.1
$\mathrm{Delta}\;Z\;(g_{Z})$	0.1	0.1	0.1	0.1
Pattern 2				
$\mathrm{Delta}\;Y\;(g_{Y})$	0.1	0.1	0.1	0.1
$\mathrm{Delta}\;Z\;(g_{Z})$	−0.1	−0.1	−0.1	−0.1
Pattern 3				
$\mathrm{Delta}\;Y\;(g_{Y})$	−0.1	−0.1	−0.1	−0.1
$\mathrm{Delta}\;Z\;(g_{Z})$	0.1	0.1	0.1	0.1
Pattern 3				
$\mathrm{Delta}\;Y\;(g_{Y})$	−0.1	−0.1	−0.1	−0.1
$\mathrm{Delta}\;Z\;(g_{Z})$	−0.1	−0.1	−0.1	0.1

**Table 3:** Relationship between the neural network outputs and the severity level.

Activated Neurons	Severity
1 0 0 0	Reduced risk
0 1 0 0	High risk
0 0 1 0	Serious risk
0 0 0 1	Critical risk

**Table 4:** Performance scores of ***NN*_2_**.

Metrics	Evaluation
Precision	0.96
Recall	0.96
F1	0.96
Accuracy	0.96

**Table 5:** Examples of base tilt pattern classification on the small-scale physical model of Figure 15 (base pattern and its variations). Distorted patterns are marked with asterisks indicating the distortion for each axis. Accuracy = 100%.

Test	Detected Angles by the Computer Model								Physical Model’s Real Pattern	Classified Pattern
	Y0	Z0	Y1	Z1	Y2	Z2	Y3	Z3		
1	0.2	0	0.2	0	0.2	0	0.2	0	0	0
2	0.9	0	0.9	0	0.9	0	0.9	0	0	0
3	1	0	0.6	0	0.4	0	0.7	0	0	0
4	1	0.1 *	0.6	0.1 *	0.4	0	0.7	0	0	0
5	1	−0.1 *	0.6	0.1 *	0.4	0	0.7	0	0	0
6	−0.2	0	−0.2	0	−0.2	0	−0.2	0	1	1
7	−0.9	0	−0.9	0	−0.9	0	−0.9	0	1	1
8	−1	0	−0.6	0	−0.4	0	−0.7	0	1	1
9	−1	0.1 *	−0.6	0	−0.4	0.1 *	−0.7	0	1	1
10	−1	−0.1 *	−0.6	0	−0.4	0.1 *	−0.7	0	1	1
11	0.2	0.2	0.2	0.2	0.2	0.2	0.2	0.2	2	2
12	0.9	0.6	0.9	0.6	0.9	0.6	0.9	0.6	2	2
13	1	0.5	0.6	0.7	0.4	1	0.7	0.8	2	2
14	0.2	0.2	0.2	0.2	0.2	0.2	0.2	0.2	2	2
15	0.8	0.6	0.6	0.5	0.4	0.3	0.4	0.4	2	2
16	0.9	−0.6	0.9	−0.6	0.9	−0.6	0.9	−0.6	2	2
17	1	−0.5	0.6	−0.7	0.4	−1	0.7	−0.8	2	2
18	1	0.5 *	0.6	−0.7	0.4	−1	0.7	−0.8	2	2
19	−0.2	−0.2	−0.2	−0.2	−0.2	−0.2	−0.2	−0.2	3	3
20	−0.9	−0.6	−0.9	−0.6	−0.9	−0.6	−0.9	−0.6	3	3
21	−1	−0.5	−0.6	−0.7	−0.4	−1	−0.7	−0.8	3	3
22	−0.2	0.2	−0.2	0.2	−0.2	0.2	−0.2	0.2	3	3
23	−0.9	0.6	−0.9	0.6	−0.9	0.6	−0.9	0.6	3	3
24	−1	0.5	−0.6	0.7	−0.4	1	−0.7	0.8	3	3
25	−1	−0.5 *	−0.6	0.7	−0.4	1	−0.7	0.8	3	3
26	−0.8	−0.1 *	−0.7	0.7	−0.6	1	0.2 *	0.8	3	3
27	−0.1	0	0.1	0	0	0	0	0	4	4
28	−0.8	0	0.7	0	0	0	0	0	4	4
29	−1.5	0	1	0	0	0	0	0	4	4
30	−1.5	0	1	0	0.1 *	0	0.1 *	0	4	4
31	−1.5	0.1 *	1	0	0.1 *	0	0.1 *	0	4	4
32	0.5	0	−0.5	0	0	0	0	0	5	5
33	1.5	0	−1	0	0	0	0	0	5	5
34	1.5	0.1 *	−1	0.2 *	0	0	0	0	5	5
35	0.6	0.2 *	−0.9	0.1 *	0	0	0	0	5	5
36	0.5	0.1 *	−1.3	0.1 *	0	0	0	0	5	5
37	0	0	0	0	0	0.5	0	−0.5	6	6
38	0	0	0	0	0	0.2	0	−0.7	6	6
39	0	0	0	0	0.1 *	0.8	0	−0.3	6	6
40	0	0	0	0	0.2 *	0.8	0	−0.3	6	6
41	0	0	0	0	0.1 *	0.8	0.2 *	−0.6	6	6
42	0	0	0	0	0	−0.5	0	0.5	7	7
43	0	0	0	0	0	−0.2	0	0.7	7	7
44	0	0	0	0	0.1 *	−0.9	0	0.4	7	7
45	0	0	0	0	0.2 *	−1.5	0	1.2	7	7
46	0	−0.1 *	0	0.2 *	0	−1	0	1	7	7
47	0	0	0.4	0.4	0.4	0.4	0	0	8	8
48	0	0	0.4	0	0	0.8	0	0	8	8
49	0	0	0.4	1	0.4	1	0	0	8	8
50	0	0	0.4	1	0.4	0.7	0	0	8	8
51	0.1 *	0.2 *	0.4	1	0.4	0.7	0	0	8	8
52	−0.6	−0.6	0	0	0	0	−0.6	−0.6	9	9
53	−0.6	−1	0	0	0	0	−0.4	−1	9	9
54	−0.6	−0.8	−0.1 *	−0.2 *	0	0	−0.4	−0.7	9	9
55	−0.6	−0.8	0.1 *	−0.2 *	0	0	−0.4	−0.7	9	9
56	−1.5	−1.5	0	0	0	0	−1.5	−1.5	9	9
57	−0.9	0.9	0	0	−0.9	0.9	0	0	10	10
58	−0.5	0.6	0	0	−0.4	0.5	0	0	10	10
59	−1.3	0.3	0	0	−1.4	0.2	0	0	10	10
60	−0.8	0.7	0.1 *	0	−0.7	1.5	0.2 *	0	10	10
61	−0.7	0.9	0.1 *	0	−0.5	1.5	0.1 *	0.2 *	10	10
62	0	0	0.7	−0.7	0	0	0.7	−0.7	11	11
63	0	0	1.3	−0.7	0	0	1.5	−0.9	11	11
64	0	0	0.4	−1.2	0	0	0.9	−0.7	11	11
65	0.2 *	−0.1 *	0.4	−1.2	0	0	0.9	−0.7	11	11
66	−0.1 *	0.2 *	0.4	−1.2	0	0	0.9	−0.7	11	11
67	−0.8	−0.8	0.8	0.8	0.8	0.8	−0.8	−0.8	12	12
68	−0.6	−1.2	0.5	1.5	0.4	0.7	−1.4	−1	12	12
69	−0.6	0.8 *	0.5	1.5	0.4	0.7	−1.4	−1	12	12
70	−0.6	1 *	0.5	1.2	0.4	0.8	−1.2	−1	12	12
71	−0.3	−0.7	0.6	−0.5 *	−0.7 *	0.3	0.3 *	−0.6	12	12
72	−0.9	0.9	0.9	−0.9	−0.9	0.9	0.9	−0.9	13	13
73	−0.8	0.2	0.4	−0.6	−0.3	0.9	0.4	−1.3	13	13
74	−1	1	0.7	−0.7	−0.1	0.1	0.9	−0.9	13	13
75	−1	−0.5 *	0.9	−1.2	−1	0.7	0.8	0.3 *	13	13
76	0.1 *	−0.5 *	0.9	−1.2	−1	0.7	0.8	−0.6	13	13
77	−0.9	0	0	0	0	0	0	0	14	14
78	−0.9	0	0	0	−0.9	0	−0.9	0	14	14
79	−0.9	0.1 *	0	0	−0.9	0.1 *	−0.9	0.1 *	14	14
80	−1	−0.1 *	0	0	−0.8	−0.2 *	−0.7	−0.1 *	14	14
81	−1.5	−0.2 *	0	0	−1	0.3 *	−1.3	−0.2 *	14	14
82	0	0	0.9	0	0	0	0	0	15	15
83	0	0	0.9	0	0.9	0	0.9	0	15	15
84	0	0	0.9	0	0.9	0.1 *	0.9	0.1 *	15	15
85	0	0	1	−0.1 *	0.8	−0.2 *	0.7	−0.1 *	15	15
86	0	0	1.5	−0.2 *	−1	0.3 *	−1.3	−0.2 *	15	15
87	0	0	0	0	0	0.8	0	0	16	16
88	0	0.9	0	0.8	0	0.9	0	0	16	16
89	0.2 *	0.9	0.1 *	0.8	0.1 *	0.9	0	0	16	16
90	−0.2 *	0.9	0.1 *	0.9	−0.1 *	1	0	0	16	16
91	0.1 *	1	0.2 *	1.2	−0.2 *	1.4	0	0	16	16
92	0	0	0	0	0	0	0	−1.3	17	17
93	0	−1.3	0	−1.3	0	0	0	−1.3	17	17
94	0.1 *	−1	0.2 *	−0.9	0	0	0.1 *	−0.7	17	17
95	−0.1 *	−1.5	−0.2 *	−1.4	0	0	−0.2	−1.2	17	17
96	0.1 *	−1.5	0.2 *	−1.4	0	0	−0.3 *	−1.2	17	17

**Table 6:** Tilt severity classification on a small-scale physical model for laboratory tests in rectangular buildings of Figure 15. Accuracy = 96%. * misclassified.

Test	Detected Angles by the Computer Model								Physical Model’s Real Severity	Classified Severity
	Y0	Z0	Y1	Z1	Y2	Z2	Y3	Z3		
1	−0.8	0	−1	0	−0.9	0.1	−0.8	0	Serious	Serious
2	1	−0.1	0.9	0	0.9	−0.1	0.9	0	Serious	Serious
3	0	−0.8	−0.1	−1	0	−1	0	−0.9	Serious	Serious
4	0.3	0	−0.2	0.1	0	0	0	0	Reduced	Reduced
5	0	0	0	0	0	−0.8	0	0.3	High	High
6	0.5	0.4	0	0	0	0	0.3	0.6	High	High
7	0	0	0.3	0.2	0.1	0	0.4	0.3	Reduced	Reduced
8	0.7	−0.8	−0.5	0.7	−0.5	0.4	−0.3	0.5	Serious	Serious
9	0	0	−0.3	−0.1	0	0	0	0	Reduced	Reduced
10	0	0	0	0	0	−0.3	0	0	Reduced	Reduced
11	0.6	0.1	0	0	0	0	0.5	0.1	High	High
12	0	0	−0.5	0.2	0	0	−0.1	0.4	High *	Reduced *
13	0.1	−0.7	−0.1	0.8	0.7	−0.6	−0.6	0.6	High	High
14	0	0	−0.9	0.1	0	0	0	0	Serious	Serious
15	1.5	−0.1	0	0	0	0	0	0	Critical	Critical
16	0	0	0	0	0.1	−0.9	0	0	Serious	Serious
17	0.3	0	0.4	0	0.2	0	0.1	0	Reduced	Reduced
18	0	−1.1	0	−0.9	0	−0.9	0	−0.1	Serious	Serious
19	0.7	0	−0.2	0	0	0	0	0	High	High
20	−0.2	0	0.7	0	0	0	0	0	High	High
21	0	0	0	0	0	−0.3	0	1	Serious	Serious
22	0	0	0	0	0	0.2	0	−0.8	High	High
23	0	0	−1.2	−0.1	−1.3	−0.1	0	0	Critical	Critical
24	0.3	0.1	0	0	0	0	1.1	0.1	Serious	Serious
25	0.1	0.3	−0.2	−0.3	−0.2	−0.1	0.3	0.5	High	High
26	0	0	−0.5	0.1	0	0	0	0	High	High
27	0	0	0	0	0.1	−1.4	0	0	Critical	Critical
28	0	0	0	0	0	0	0	0.5	High	High
29	−0.1	−0.8	0	−0.5	−0.1	−0.6	0.1	−0.5	Serious	Serious
30	1.3	1.1	−1.1	−0.9	−1.1	−1.0	1.3	1.1	Critical	Critical

## Data Availability

The data presented in this study are available on request from the corresponding author.

## Appendix A

Figure A1 presents an example of a building in Mexico City with a tilt greater than 1° that is under supervision; its inclination is visually appreciated compared to the adjacent building that does not have a relevant tilt. Figure A2 shows the palace of fine Arts in Mexico City, which has presented differential subsidence (settlement) with associated inclinations for years.

Figure A3a presents an example of the interface for permanent monitoring. It allows information processing to visualize the angles in real time, the time at which the event occurred, the detected tilt pattern, and its severity. When the system detects a pattern, the machine learning models define the activation of emerging alarms; for example, Figure A3b provides the most relevant information.

## Appendix B

Table A1 presents additional examples of tilt severity classification. Two of the 50 tests were misclassified (numbers 22 and 41). These are excellent results considering the mathematically subtle differences, which can be infrequent in real buildings.

**Table A1:** Additional examples of tilt severity classification tests on the physical model at laboratory scale of Figure 15. Accuracy = 96%. * misclassified.

Test	Detected Angles by the Computer Model								Physical Model’s Real Severity	Classified Severity
	Y0	Z0	Y1	Z1	Y2	Z2	Y3	Z3		
1	−0.7	0	−0.9	0	−0.8	0.1	−0.8	0	Serious	Serious
2	1	−0.2	0.8	0.1	0.9	−0.1	0.9	0	Serious	Serious
3	0	−0.7	−0.2	−1	0	−0.8	0	−0.9	Serious	Serious
4	−0.9	−0.8	−0.2	−0.9	0.3	−0.9	−0.5	−0.9	Serious	Serious
5	0.1	0	−0.2	0.2	0	0	0	0	Reduced	Reduced
6	0.2	0	−0.1	0.2	0	0	0	0	Reduced	Reduced
7	0	0	0	0	0	−0.8	0	0.4	High	High
8	0.4	0.5	0	0	0	0	0.6	0.3	High	High
9	0.5	0.6	0	0	0	0	0.4	0.5	High	High
10	0	0	0.2	0.3	0.1	0	0.3	0.4	Reduced	Reduced
11	0	0	0.1	0.2	0.1	0	0.2	0.3	Reduced	Reduced
12	0.8	−0.7	−0.6	0.7	−0.4	0.5	−0.3	0.5	Serious	Serious
13	0.7	−0.6	−0.5	0.6	−0.3	0.4	−0.3	0.5	Serious	Serious
14	0	0	−0.1	−0.3	0	0	0	0	Reduced	Reduced
15	0	0	0	0	0	−0.2	0	0	Reduced	Reduced
16	0	0	0.1	0	0	−0.2	0	0	Reduced	Reduced
17	0.6	0.1	0	0	0	0	0.6	0.1	High	High
18	0.1	0.5	0	0	0	0	0.1	0.5	High	High
19	0.1	0.5	0	0	0	0	0.1	0.5	High	High
20	0.1	−0.5	0	0	0	0	0.1	−0.5	High	High
21	−0.5	0.1	0	0	0	0	−0.5	0.1	High	High
22	0	0	0.4	0.2	0	0	0.2	0.5	High *	Reduced *
23	0	0	−0.8	0.2	0	0	−0.2	0.6	High	High
24	0.7	0.2	0	0	0.2	0.6	0	0	High	High
25	−0.8	−0.3	0	0	−0.1	−0.5	0	0	High	High
26	−0.1	0.7	0.1	−0.8	−0.7	0.6	0.6	−0.6	High	High
27	−0.1	0.6	0.1	−0.7	−0.6	0.5	0.5	−0.5	High	High
28	−0.2	0.5	0.2	−0.6	−0.5	0.4	0.4	−0.4	High	High
29	0	0	−1	0.2	0	0	0	0	Serious	Serious
30	0.9	0.1	0	0	0	0	0	0	Serious	Serious
31	−0.9	−0.1	0	0	0	0	0	0	Serious	Serious
32	−1.5	0.1	0	0	0	0	0	0	Critical	Critical
33	0	0	1.5	−0.1	0	0	0	0	Critical	Critical
34	0	0	0	0	−1.5	0.1	0	0	Critical	Critical
35	0	0	0	0	0	0	0.1	1.5	Critical	Critical
36	0	0	0	0	0.1	−0.9	0	0	Serious	Serious
37	0	0.3	0	0.4	0	0.2	0	0.1	Reduced	Reduced
38	0	1	0	0.9	0	0.9	0	0.1	Serious	Serious
39	−0.7	0	0.2	0	0	0	0	0	High	High
40	0.2	0	−0.7	0	0	0	0	0	High	High
41	0	−0.1	0	−0.1	0.1	−0.2	0.1	−0.9	Serious *	High *
42	0	0	0	0	0	−0.2	0	0.8	High	High
43	0	0	1.2	0.1	1.3	0.1	0	0	Critical	Critical
44	−0.3	−0.1	0	0	0	0	−1.1	−0.1	Serious	Serious
45	−0.1	−0.3	0.2	0.3	0.2	0.1	−0.3	−0.5	High	High
46	0	0	0.5	−0.1	0	0	0	0	High	High
47	0	0	0	0	−0.1	1.4	0	0	Critical	Critical
48	0	0	0	0	0	0	0	−0.5	High	High
49	0.1	0.8	0	0.5	0.1	0.6	−0.1	0.5	Serious	Serious
50	−1.3	−1.1	1.1	0.9	1.1	1.0	−1.3	−1.1	Critical	Critical
